# Impact of Periodontal Diseases on Health-Related Quality of Life of Users of the Brazilian Unified Health System

**DOI:** 10.1155/2013/150357

**Published:** 2013-12-18

**Authors:** Pamella Valente Palma, Paula Liparini Caetano, Isabel Cristina Gonçalves Leite

**Affiliations:** ^1^School of Dentistry, Institutional Programs for Scientific Start-Up Grants (XX PIBIC/UFJF), Federal University of Juiz de Fora (UFJF), 36036-900 Juiz de Fora, MG, Brazil; ^2^Public Health Department of the School of Medicine, Federal University of Juiz de Fora, Street José Lourenço Kelmer, Campus Universitário-São Pedro, 36036-900 Juiz de Fora, MG, Brazil

## Abstract

*Objective.* This study assessed the impact of periodontal diseases on health-related quality of life of adult users of the Brazilian Unified Health System. *Study Design.* A cross-sectional study was conducted on an outpatient basis. The sample included 151 adults treated in the Periodontics section at Dental Specialty Centres of Juiz de Fora (Minas Gerais, Brazil). The *Oral Health Impact Profile* (OHIP-14) measured the impact of periodontal disease on quality of life. Participants were interviewed to obtain self-perception of general and oral health and socioeconomic data, and dental records were consulted to obtain periodontal status data. The values of central tendency of the OHIP-14 were compared with socioeconomic, demographic, and self-reported health predictors using nonparametric tests. The final analysis was performed using multiple linear regressions. *Results.* The results showed that psychological discomfort and physical disability exhibited a negative impact. The following variables can explain approximately 27% of the impact of oral health conditions on health-related quality of life in this group: periodontal disease, self-perceived oral health, and the need to use or replace dental prosthesis. *Conclusion.* The need for prosthetic rehabilitation and worse periodontal status are associated with health-related quality of life, which can be predicted by the self-perception of health.

## 1. Introduction

Diseases that affect the teeth are as old as man himself, but epidemiological surveys of oral health conditions began in the 1960s. An experimental study on gingivitis in humans [1] showed that the build-up of bacterial plaque led to the development of gingival inflammation (gingivitis), and its removal eliminated the lesions. These systematic observations have become an important tool for determining the factors that cause disease in individuals with similar characteristics, and these observations have contributed to the prevention and treatment of different diseases. Some common chronic diseases of the oral cavity and their consequences influence an individual's general and collective well-being.

The worldwide prevalence of periodontal disease is 5–20% in the adult population. Periodontitis is the second largest oral health problem, affecting 10–15% of the world's population [2]. The presence of dental calculus and bleeding tend to be more common in 12-year olds and adolescents. The most severe forms of periodontal disease significantly affect adults (35–44 years old) with a prevalence of 19%. Gum problems in the elderly have a reduced impact at the population level due to the reduced number of present teeth [3].

Therefore, periodontal disease is highly prevalent and interferes with health-related quality of life in many ways, including the physical aspect, masticatory function, appearance, and interpersonal relationships [4].

Many quality of life indices to assess health in populations with chronic diseases, such as periodontal disease, allow the researcher to determine the impact of health care, especially in the absence of a cure [5].

The *Oral Health Impact Profile* (OHIP-14) is a widely used index for the measurement of oral health on the quality of life in patients with oral diseases. This index is a good indicator of individual perceptions and feelings of their own oral health and their expectations regarding dental treatment and services [6].

The present study assessed the impact and severity of periodontal disease on health-related quality of life in adult users of the Brazilian Unified Health System (Sistema Único de Saúde—SUS).

## 2. Materials and Method

The study was a cross-sectional study of patients diagnosed and treated in the Dental Specialty Centres (Centros de Especialidades Odontológicas—CEOs) of Juiz de Fora. The Ethics Committee on Human Research of PROPESQ/UFJF approved this study (Opinion no. 337.177).

The study sample included adult users of the CEOs who were treated in the Periodontics section of the Sistema Único de Saúde (SUS) network, Juiz de Fora, state of Minas Gerais (MG), Brazil, who underwent basic clinical dental care and signed an informed consent form. The Unified Health System is the Brazilian public healthcare system that was created in 1988 and provides universal, equal, full, and free care to approximately 75.5% of the population who has no other access to healthcare services. The CEOs were created in 2004 and offer specialised care in periodontics, endodontics, minor oral surgery, and oral diagnosis, and care for patients with special needs.

The following exclusion criteria were used: patients classified as code zero using Periodontal Screening and Recording (PSR); patients unable to interpret or answer the questions, such as patients with neurological or cognitive disorders; users of complete dentures; and patients with painful dental conditions.

Participants were interviewed to obtain identification, self-perceptions of general and oral health (including the need to use or replace prosthesis) and socioeconomic data. Periodontal status was obtained from standardised dental records and data from the initial clinical examination. The examinations conducted by the four periodontists were periodically calibrated (last interexaminer agreement of 0.81 and intraexaminer agreement of 0.88 for the PSR). Dental status was also obtained from odontograms in the analysed dental reports. All patients answered the validated *Oral Health Impact Profile* (OHIP-14) adapted to Brazilian Portuguese [6]. Slade and Spencer developed the original OHIP [7]. The Portuguese version of the OHIP-14 showed good psychometric properties similar to the original instrument. Therefore, the present study did not validate the OHIP because it has been validated in a population with similar sociodemographic and cultural characteristics. However, questionnaires were reapplied within seven days in 10 individuals in the pilot study to increase the reliability of results. The test-retest reliability was analysed using Pearson's correlation coefficient (0.83; *P* < 0.01) and Cronbach's alpha test (0.92), and the results showed stability and internal consistency. These results demonstrated that the examiner properly applied the questionnaire.

Data collection occurred between August and September 2013. The sample was estimated from the maximum number of first medical appointments in the three CEOs during one month (a total of five specialists X six individuals in the first appointment/week). The estimated sample size included 147 individuals assuming an average prevalence of 85% of periodontal disease in a total population of 360 available patients plus 20% for potential participation refusals.

A total of 151 patients were approached during this period, and one patient refused to participate. Therefore, the study included 150 patients diagnosed with periodontal disease. From this group, 38 patients were diagnosed with gingivitis, and 112 patients were diagnosed with periodontitis.

The criterion adopted to differentiate individuals with periodontitis and gingivitis relied on the clinical diagnosis of periodontists from the SUS network using the PSR defined by the American Dental Association and American Academy of Periodontology [8].

The descriptive analysis of OHIP-14 used dichotomised answers to quantify each dimension. The answers “often” and “always” had an* impact*, but answers of “sometimes” “rarely,” and “never” had* no impact*.

The maximum final score of the overall OHIP-14 was 56 points (two questions per domain with a maximum score of four points equals eight per domain for seven domains).

All data scores were subjected to the Kolmogorov-Smirnov test. The domains and sums were not normally distributed (*P* > 0.05). Therefore, a bivariate analysis was performed using the nonparametric Mann-Whitney and Kruskal-Wallis tests, which analyse the relationship between selected independent variables (e.g., oral morbidity, self-perception of general and oral health, self-reported skin colour, gender, socioeconomic status, professional occupation, marital status, education level, and age of the respondents) and the dependent variable (i.e., impact of oral health on quality of life). Multivariate analysis was conducted to identify predictors of the OHIP-14 and control for possible confounding variables. Multiple linear regression analysis was performed on the variables that were significantly associated with the overall OHIP-14 in the bivariate analysis. Variables with *P*-values < 0.05 were retained in the final model.

## 3. Results

The sample included 62% females with a mean age of 47 years (SD = 13.5). A total of 39% of the sample had less than eight years of education, and 33% had a median household income of 2,631.20 dollars. The DMFT (decayed, missing, and filled tooth) index was 15.9 with a mean of 5% missing teeth and 11% restored teeth in the composition of the indicator. Three cases received a PSR code one; 35 cases received a code two; 104 cases received a code three; and eight cases were classified as code four.

The psychological discomfort and physical disability OHIP-14 domains displayed the same frequency (82%) (Table 1).

No association between gender and the overall OHIP-14 was found (*P* = 0.097), but associations with physical pain (*P* = 0.005) and psychological disability (*P* = 0.007) were identified. Age, marital status, education level, and professional occupation were not significantly associated with the overall OHIP-14 (*P* = 0.215, *P* = 0.365, *P* = 0.788, and *P* = 0.200, resp.) or specific domains. Socioeconomic class was associated with social disability (*P* = 0.027). Self-reported skin colour was associated with psychological disability (*P* = 0.043) (Table 2).

The association between self-perceived general health and functional disability (*P* = 0.051) was statistically significant. Individuals who perceived their general health as poor showed higher quality of life scores due to oral conditions. The association between self-perceived oral health and the overall OHIP-14 and different domains was statistically significant (*P* < 0.05). Individuals who perceived their oral health as poor showed higher quality of life scores due to oral conditions. The self-reported need to use complete or partial prosthesis or replace the prosthesis in use remained significantly associated with the overall OHIP-14 (*P* < 0.001) and all domains except functional limitation. Dentition status was statistically associated with functional limitation (*P* = 0.032) and disability (*P* = 0.012). Periodontal disease was significantly associated with psychological discomfort (*P* = 0.029) and physical disability (*P* = 0.029) and the overall OHIP-14 (*P* = 0.017) (Table 3).

Table 4 shows the results of the regression analysis. Self-perception of oral health, the need to replace or use a prosthesis, and periodontal disease remained significant after fitting. These results indicate that the greatest impact of variables related to oral health on OHIP-14 was socioeconomic conditions. Moreover, the need for prosthetic rehabilitation and personal judgment were more significant than the periodontal status. The final model explains 27% of the variability of the final OHIP-14 score in the sample, which indicates the existence of other explanatory variables that were not measured in this study.

## 4. Discussion

Few Brazilian studies associate periodontal disease with indicators of quality of life related to oral health, but this issue has been investigated in specific population groups, such as the elderly, pregnant women, or diabetics. This study extended the ability to generalise the data to the Brazilian population who depends on the public healthcare system.

Socioeconomic factors were closely linked to oral health-related quality of life. Sociodemographic factors, such as gender, income, education level, and age should be controlled in measurements of the impact of oral health on quality of life to obtain a sample as homogenous as possible [9, 10].

The present study included a greater number of women in the sample. This observation is a common finding in institutional samples that may be explained by cultural moorings that hinder the adoption of self-care practices in males. Men are seen as manly, strong, and invulnerable, and the search for preventive healthcare could be associated with weakness, fear, and insecurity [11].

Previous findings of gender in the studies that used the OHIP-14 demonstrated a negative impact of being female on health-related quality of life [11–13], which is similar to the present study in which physical pain (*P* = 0.005) and psychological disability (*P* = 0.007) were significantly associated with being a female.

Among the potential variables that negatively influence the various measurements of oral health-related quality of life, the clinical indices were reported most frequently among patients with periodontal disease. Socioeconomic factors, such as age and gender [13], may influence clinical characteristics because these factors determine lifestyle, housing, access to products, oral hygiene conditions, access to healthcare services, and education level. The results of this study exemplify this situation in which physical pain and physical disability showed an impact of 78.7% and 82%, respectively. The variables that were potentially associated with these domains, namely socioeconomic factors and gender, remained significant only in the bivariate analysis in the present study.

The need for the use or replacement of prosthesis negatively impacted oral health-related quality of life (*P* = 0.007). This same result was found in a previous study in Brazil [14] and may be explained by the oral health condition of the Brazilian population and public policies that prioritise children. Adult dental care access provided by state was considered a mutilating practice. The impact of prosthetic needs is not measured in studies in developed countries, which suggests that this age group requires more attention.

The majority of previous studies concluded that periodontal diseases are associated with a worse health-related quality of life, and this impact increases with disease severity. This study confirmed the significant association between more severe periodontal disease (periodontitis) with worse scores in psychological discomfort (*P* = 0.029) and physical disability (*P* = 0.029) and the overall OHIP-14 score (*P* = 0.017), which were controlled by sociodemographic variables.

A strong correlation was observed between the single question on self-perceived oral health and the OHIP-14. Therefore, this indicator captures the need reported by the individual and displays a scenario that is closer to the individual's actual oral health status. Self-perception of oral health and general health showed the greatest association with the overall OHIP-14 (*P* < 0.001 in the overall OHIP-14). These findings corroborate previous studies [15, 16]. Self-perceived oral health remained associated with the overall OHIP-14 after fitting for other variables in the multivariate regression.

The present study exhibits some limitations, such as the classification of periodontal severity using only two levels and the inclusion of patients who were excluded in the PSR analysis. However, these inclusions reflect the condition of the Brazilian adult population.

The self-reported need for prosthetic rehabilitation and worse periodontal status are associated with oral health-related quality of life. The perception of the latter may be predicted by its self-perception. These data confirm that subjective indicators are important in the analysis of individually reported needs.

## 5. Conclusions

The results of the present study may be useful for planning other work involving the theme of quality of life in periodontal diseases and for the improvement and further development of public policies in the area, as well as improving actions aimed at promoting health and awareness of the importance of proper oral hygiene habits, to achieve better control of the disease.

## Figures and Tables

**Table 1:** Distribution of patients treated in the Periodontics section at Dental Specialty Centres (Centros de Especialidades Odontológicas), Juiz de Fora, 2013, according to the frequency of impact per OHIP-14 domains.

Oral health dimension	No impact *n* (%)	With impact *n* (%)
Functional limitation	81 (54)	69 (46)
Physical pain	32 (21.3)	118 (78.7)
Psychological discomfort	27 (18)	123 (82)
Physical disability	27 (18)	123 (82)
Psychological disability	40 (26.7)	110 (73.3)
Social disability	98 (65.3)	52 (34.7)
Disability	92 (61.3)	58 (38.7)

**Table 2:** Mean, standard deviation, and *P*-value (Mann-Whitney and Kruskal-Wallis) of socioeconomic and demographic variables per domain and overall OHIP-14 of patients treated in the Periodontics section, Dental Specialty Centres (Centros de Especialidades Odontológicas), Juiz de Fora, 2013.

Variable	Mean per domain (SD)							
	1	2	3	4	5	6	7	Total
Gender								
Male	0.41 (0.52)	0.74 (0.52)	0.84 (0.55)	0.84 (0.55)	0.56 (0.50)	0.41 (0.61)	0.35 (0.52)	4.16 (2.58)
Female	0.46 (0.55)	0.98 (0.54)	0.95 (0.54)	0.95 (0.54)	0.80 (0.53)	0.33 (0.51)	0.39 (0.53)	4.88 (2.68)
*P*-value	0.72	**0.005**	0.188	0.188	**0.007**	0.643	0.620	0.097
Age								
18–47 years old	0.44 (0.51)	0.93 (0.51)	0.98 (0.55)	0.98 (0.55)	0.77 (0.51)	0.39 (0.54)	0.39 (0.56)	4.89 (2.54)
Over 47 years old	0.43 (0.57)	0.85 (0.56)	0.85 (0.54)	0.85 (0.54)	0.66 (0.54)	0.34 (0.56)	0.36 (0.50)	4.35 (2.74)
*P*-value	0.742	0.455	0.128	0.128	0.200	0.494	0.911	0.215
Self-reported skin colour								
White	0.46 (0.56)	0.84 (0.57)	0.91 (0.56)	0.91 (0.56)	0.62 (0.52)	0.33 (0.51)	0.34 (0.50)	4.42 (2.69)
Nonwhite	0.42 (0.52)	0.94 (0.50)	0.91 (0.54)	0.91 (0.54)	0.80 (0.53)	0.39 (0.59)	0.41 (0.55)	4.79 (2.63)
*P*-value	0.682	0.325	0.956	0.956	**0.043**	0.818	0.523	0.403
Marital status								
Nonmarried	0.54 (0.59)	0.81 (0.55)	0.97 (0.55)	0.97 (0.55)	0.77 (0.52)	0.37 (0.54)	0.40 (0.54)	4.83 (2.77)
Married	0.34 (0.47)	0.97 (0.52)	0.86 (0.54)	0.86 (0.54)	0.65 (0.54)	0.36 (0.56)	0.35 (0.52)	4.38 (2.54)
*P*-value	0.32	0.68	0.174	0.174	0.214	0.789	0.461	0.365
Education level								
Elementary	0.44 (0.57)	0.90 (0.53)	0.88 (0.58)	0.88 (0.58)	0.73 (0.57)	0.38 (0.57)	0.38 (0.55)	4.58 (2.87)
Secondary + higher	0.43 (0.50)	0.88 (0.56)	0.95 (0.50)	0.95 (0.50)	0.68 (0.48)	0.35 (0.53)	0.37 (0.50)	4.63 (2.41)
*P*-value	0.800	0.983	0.475	0.475	0.563	0.849	0.873	0.788
Gross income in dollars/month—ABEP classification								
USD 12,054. 30 + USD 6,104.20	0.38 (0.50)	0.86 (0.55)	0.85 (0.60)	0.85 (0.60)	0.60 (0.50)	0.23 (0.49)	0.32 (0.48)	4.08 (2.58)
USD 3,875. 50 + USD 2,631.20 + USD 1,784.80	0.47 (0.56)	0.91 (0.54)	0.94 (0.52)	0.94 (0.52)	0.76 (0.54)	0.42 (0.57)	0.40 (0.55)	4.84 (2.67)
*P*-value	0.390	0.672	0.532	0.532	0.132	**0.027**	0.530	0.123
Professional occupation								
Work	0.47 (0.54)	0.91 (0.48)	0.89 (0.53)	0.89 (0.53)	0.71 (0.50)	0.40 (0.57)	0.42 (0.58)	4.71 (2.68)
Does not work	0.48 (0.58)	1.01 (0.61)	1.10 (0.51)	1.10 (0.51)	0.81 (0.57)	0.44 (0.56)	0.30 (0.39)	5.23 (2.38)
Retired	0.37 (0.52)	0.79 (0.59)	0.85 (0.57)	0.85 (0.56)	0.64 (0.56)	0.25 (0.51)	0.33 (0.50)	4.09 (2.72)
*P*-value	0.461	0.195	0.166	0.166	0.501	0.173	0.717	0.200

Significant (*P*-value < 0.005).

**Table 3:** Mean and *P*-value (Mann-Whitney) of oral morbidity and self-perception of health per domain and overall OHIP-14 of patients treated in the Periodontics section, Dental Specialty Centres (Centros de Especialidades Odontológicas), Juiz de Fora, 2013.

Variable	Mean per domain (SD)							
	1	2	3	4	5	6	7	Total
Self-perceived oral health								
Poor	0.55 (0.57)	0.95 (0.52)	0.93 (0.56)	0.93 (0.56)	0.79 (0.53)	0.38 (0.60)	0.39 (0.57)	4.92 (2.79)
Good	0.37 (0.50)	0.85 (0.55)	0.90 (0.54)	0.90 (0.54)	0.65 (0.53)	0.35 (0.52)	0.36 (0.50)	4.39 (2.56)
*P*-value	**0.006**	**0.012**	**<0.001**	**<0.001**	**<0.001**	**0.01**	**<0.001**	**<0.001**
Self-perceived general health								
Poor	0.52 (0.57)	0.97 (0.51)	1.02 (0.50)	1.02 (0.50)	0.84 (0.52)	0.46 (0.60)	0.47 (0.55)	5.30 (2.56)
Good	0.23 (0.38)	0.67 (0.58)	0.63 (0.57)	0.63 (0.57)	0.35 (0.39)	0.10 (0.25)	0.13 (0.39)	2.75 (1.93)
*P*-value	**0.051**	0.272	0.690	0.690	0.143	0.964	0.963	0.237
Self-reported need to use complete or partial prosthesis, or to replace that in use								
Yes	0.47 (0.54)	1.03 (0.51)	1.06 (0.49)	1.06 (0.49)	0.85 (0.55)	0.46 (0.61)	0.47 (0.55)	5.39 (2.63)
No	0.40 (0.54)	0.71 (0.53)	0.72 (0.56)	0.72 (0.56)	0.52 (0.44)	0.24 (0.44)	0.25 (0.47)	3.57 (2.32)
*P*-value	0.380	**<0.001**	**<0.001**	**<0.001**	**<0.001**	**0.032**	**0.007**	**<0.001**
Dentition status								
With teeth	0.41 (0.53)	0.91 (0.54)	0.92 (0.55)	0.92 (0.55)	0.69 (0.52)	0.34 (0.54)	0.34 (0.50)	4.54 (2.62)
Toothless	0.77 (0.59)	0.68 (0.56)	0.82 (0.46)	0.82 (0.46)	0.88 (0.64)	0.62 (0.63)	0.77 (0.67)	5.35 (3.07)
*P*-value	**0.032**	0.143	0.368	0.368	0.277	0.093	**0.012**	0.379
Periodontal disease								
Gingivitis	0.30 (0.50)	0.81 (0.59)	0.74 (0.58)	0.74 (0.58)	0.58 (0.57)	0.29 (0.46)	0.26 (0.50)	3.74 (2.68)
Periodontitis	0.49 (0.55)	0.92 (0.52)	0.97 (0.52)	0.97 (0.52)	0.75 (0.51)	0.39 (0.58)	0.41 (0.53)	4.90 (2.59)
*P*-value	0.063	0.407	**0.029**	**0.029**	0.062	0.626	0.068	**0.017**

Significant (*P*-value < 0.005).

**Table 4:** Final result of the multiple linear regressions for patients treated in the Periodontics section at Dental Specialty Centres (Centros de Especialidades Odontológicas), Juiz de Fora, 2013.

Variable	*β*_ adjusted_	*P*
Gender	−0.108	0.138
Periodontal disease	0.110	**0.058**
Professional occupation	−0.115	0.126
Gross income in dollars/month—ABEP classification	0.075	0.309
Self-perceived oral health	0.321	**<0.001**
Need to use or replace prosthesis	−0.216	**0.007**
Dentition status	0.031	0.673

Significant (*P*-value < 0.005).
